# Urinary Chemokines in the Diagnosis and Monitoring of Immune Checkpoint Inhibitor-Associated Nephritis

**DOI:** 10.3390/ijms27031240

**Published:** 2026-01-26

**Authors:** Francisco Gomez-Preciado, Laura Martinez-Valenzuela, Paula Anton-Pampols, Xavier Fulladosa, María Jove, Ernest Nadal, Josep María Cruzado, Joan Torras, Juliana Draibe

**Affiliations:** 1 Department of Nephrology, Bellvitge University Hospital, Bellvitge Biomedical Research Institute (IDIBELL), 08907 Hospitalet de Llobregat, Barcelona, Spain; fgomezp@bellvitgehospital.cat (F.G.-P.); xfulladosa@bellvitgehospital.cat (X.F.); jmcruzado@bellvitgehospital.cat (J.M.C.); jbordignon@bellvitgehospital.cat (J.D.); 2 Facultat de Medicina i Ciències de la Salut, Universitat de Barcelona (UB), 08036 Barcelona, Spain; 3 Department of Nephrology, Can Ruti Hospital, 08916 Badalona, Barcelona, Spain; pantonp.germanstrias@gencat.cat; 4 Department of Medical Oncology, Catalan Institute of Oncology, Bellvitge Healthcare Campus Comprehensive Cancer Center, 08908 Hospitalet de Llobregat, Barcelona, Spain; 5 Preclinical and Experimental Research in Thoracic Tumors (PRETT), Bellvitge Biomedical Research Institute (IDIBELL), 08908 Hospitalet de Llobregat, Barcelona, Spain

**Keywords:** nephritis, checkpoint, biomarkers, immunology

## Abstract

Immune checkpoint inhibitors are essential treatments for many oncologic diseases, but with well-known immune-related adverse events, such as acute interstitial nephritis (ICI-AIN). We investigated novel potential biomarkers that could assist in the diagnosis and follow-up of this condition and that are related to the active pathogenic pathways involved. We measured urinary soluble PD-1, PD-L1 and PD-L2, as well as chemokines CXCL5, CXCL9, CXCL10, CXCL11, CCL2, CCL3, CCL5 and cytokines IL-6 and IL-12p70 performing a Luminex assay in urine from patients with ICI-AIN (n = 35) and compared them with patients with AIN from other causes (non-ICI AIN) (n = 29) and ATN (n = 26). We found that CXCL5, CXCL9, CXCL10, CXCL11, CCL5 and IL-6 were higher in patients with ICI-AIN than in those with ATN, and all of them but CXCL9 and IL-6 were also higher in patients with ICI-AIN compared with non-ICI AIN. We also determined these molecules at follow-up for ICI-AIN patients (40 samples from 22 patients) and found that concentrations of CXCL9, CXCL10, CXCL11 and CCL2 decreased after treatment. The decrease of CXCL9 and CXCL10 correlated with greater kidney function recovery at one-year follow-up. These molecules could serve as noninvasive biomarkers and may aid fine patient monitoring.

## 1. Introduction

Immune checkpoint inhibitors (ICIs) are targeted antineoplastic drugs that have improved treatment outcomes in several malignancies and have expanded their clinical indications in recent years [1]. They act in the core of T cell receptor (TCR) signaling. When the T cell receptor (TCR) complex recognizes antigenic peptides presented by the major histocompatibility complex (MHC) on antigen-presenting cells (APCs) if co-signaling pathways (such as CD28 binding to their ligands (CD80/CD86)) are active, T cell activation happens. However, when the checkpoint inhibitor pathway is active, it dampens this activation. The transmembrane molecules programmed cell death ligand 1 (PD-L1) and 2 (PD-L2), expressed mainly on APCs, interact with programmed death 1 (PD-1), which is expressed in T, B and NK cells. This interaction induces anergy in effector cells, a mechanism essential for preventing autoimmunity and excessive immune activation. As a means of evading immune surveillance, cancer cells can overexpress PD-L1 and PD-L2, thereby promoting immune tolerance. ICIs block this inhibitory pathway, restoring the antitumor activity of the immune system [2,3].

The blockade of inhibitory pathways—which consequently leads to overactivation of immune cells—can result in immune-related adverse events (irAEs) that may affect virtually all organs, including the kidneys [4]. ICI-associated acute kidney injury (ICI-AKI) has been reported with an incidence of 2–5% of patients treated with ICI [5], most commonly presenting as acute interstitial nephritis (ICI-AIN) [6]. Histologically, AIN consists of immune-mediated infiltration of the renal tubulointerstitial compartment [7], and may be triggered by certain drugs, infections, autoimmune diseases or other unidentified factors. Its pathophysiology is not yet fully understood; however, the condition is fundamentally rooted in a cell-mediated immune reaction directed against exogenous or endogenous antigens that can act as haptens when bound to tubular basement membrane components. The mechanisms underlying ICI-AIN may differ from those of non-ICI AIN, as suggested by differences in immunochemistry [8], urine proteomics [9], and gene expression [10]. For the development and maintenance of these local immune phenomena, chemokines such as the C-X-C and C-C subfamilies are indispensable as they are responsible for the migration of inflammatory cells to the kidney tissue. Some authors have hypothesized about the relationship of these chemokines with tertiary lymphoid structures (TLS) [9]. TLS are organized aggregates of immune cells composed by a B-cell germinal center surrounded by T cells and dendritic cells, resembling secondary lymphoid organs. These structures have been identified in solid tumors and are associated with better cancer prognosis and improved responses to ICI therapy [11], and may be the structures synthesizing chemokines. In any case, AIN is a heterogeneous group of diseases in which different inflammatory pathways converge depending on the cause [12].

Acute kidney injury (AKI) in cancer patients treated with ICIs poses a major diagnostic challenge. Obstructive, functional, paraneoplastic, pharmacologic and other possible etiologies frequently coexist in the same patient [6,13,14]. Even when tubulointerstitial compartment compromise is suspected, both acute tubular necrosis (ATN) and AIN are probable, so a definitive diagnosis of ICI-AIN typically requires a kidney biopsy, which remains the gold standard. However, many of these patients are not suitable candidates due to contraindications. Furthermore, kidney function recovery is particularly important in cancer patients, as specific estimated glomerular filtration rates (eGFR) thresholds determine eligibility for certain therapeutic options [15]. Early diagnosis and treatment of AKI are therefore important to optimize renal outcomes.

Interest in novel, non-invasive biomarkers of ICI-AIN for the early diagnosis and monitoring of ICI-AIN has increased in recent years [10,16,17,18,19,20,21,22]. In a previous study, our group demonstrated that urinary soluble (us) PD-1 reliably distinguished AIN from ATN, particularly in cancer patients, and that this biomarker was correlated positively with tubular PD-L1 expression on immunostaining [23].

Therefore, the aims of our study were: (1) to confirm the utility of the soluble fraction of immune checkpoint molecules in distinguishing AIN from ATN, as suggested in our previous study; (2) to evaluate whether urinary concentrations of chemokines differ among patients with ICI-AIN, non-ICI AIN, and ATN, as well as to assess their diagnostic performance in comparison with immune checkpoint molecules; and (3) to determine their potential utility as monitoring tools during follow-up.

## 2. Results

### 2.1. Baseline Characteristics of the Cohort

We recruited 26 patients with ATN and 64 patients with AIN. Baseline characteristics of the patients are shown in Table 1.

A greater proportion of patients in the AIN group had eosinophilia compared with patients with ATN (*p* = 0.0045). Furthermore, patients with AIN had more leukocytes per microliter in urine (*p* = 0.02) and higher c-reactive protein (*p* = 0.015) than patients with ATN.

Of the 64 individuals with AIN, the etiology in 35 patients was ICI (ICI-AIN), whereas in the other 29 it was other (non-ICI AIN). Baseline characteristics of these subgroups are summarized in Supplementary Table S1. Of these patients, two (3.1%) were on low dose prednisone treatment before the AKI, and maintained that dose until baseline sample determination. A total of 43 of 64 (67.19%) patients were receiving no corticosteroids before sampling and 19 of 64 patients (29.69%) had high dose corticosteroid started before sampling due to clinical reasons. The median of days between corticosteroid initiation and sampling in these patients was three (IQR 1–7).

### 2.2. Checkpoint Pathway Molecules as Diagnostic Biomarkers

**Figure 1:**
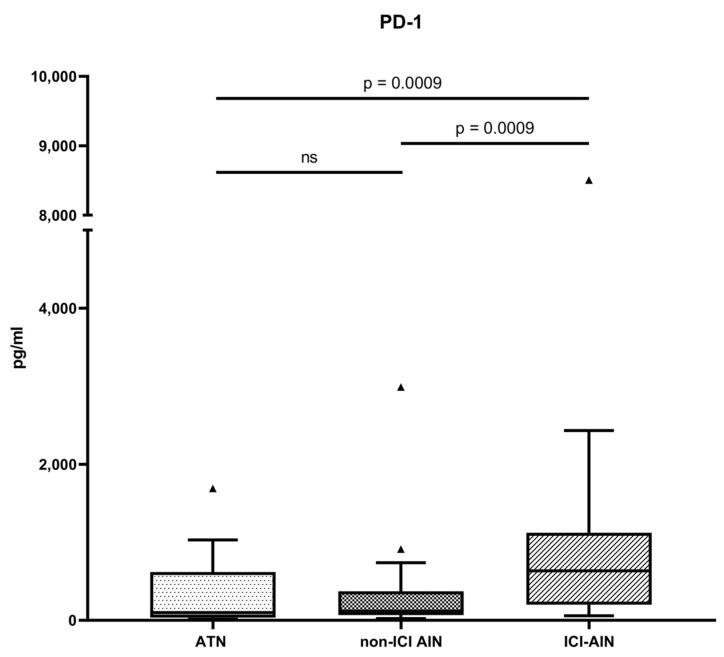
usPD-1 concentration in different study groups. *p* adjusted for multiple comparisons.

First, we investigated differences in the soluble fraction of immune checkpoint pathway molecules in urine across the groups. The highest levels of usPD-1 were observed in patients with ICI-AIN (633.7 [201.5–1121] pg/mL) compared with patients with ATN (98.45 [35.96–617.9] pg/mL; *p* (adjusted) = 0.0009) with a Hodges–Lehmann median difference of 361.1 (95% CI: 125.8 to 607.7) or non-ICI AIN (118.6 [67.18–371.3] pg/mL; *p* (adjusted) = 0.0009) with a Hodges–Lehmann median difference of 398.4 (95% CI: 143.4 to 609) (Figure 1).

No significant differences in usPD-L1 and usPD-L2 levels were observed among the groups.

Thereafter, we plotted the Receiver Operating Characteristic (ROC) curve of usPD-1 to evaluate the performance of the biomarker in distinguishing ICI-AIN from ATN. The ROC curve yielded an area under curve (AUC) of 0.79 (95% CI: 0.6529–0.9032, *p* (adjusted) = 0.0009). We identified 129.3 pg/mL as the optimal cutoff value for the usPD-1 using the Youden method, maximizing sensitivity and specificity. This cutoff was associated with a sensitivity of 97.17% and a specificity of 61.54% with a 2.52 PLR in our cohort.

### 2.3. Chemokines and Inflammation-Related Molecules as Diagnostic Biomarkers

We then evaluated whether levels of TLS-related and inflammation-associated molecules differed across the various tubulointerstitial conditions and assessed their potential as diagnostic biomarkers.

Members of the C-X-C motif chemokine ligand (CXCL) family—CXCL5, CXCL9, CXCL10, and CXCL11—and C-C motif chemokine ligand (CCL) 5 were significantly elevated in ICI-AIN patients compared with those with ATN, and most were also higher than in non–ICI AIN. No significant differences were observed for CCL2 or CCL3.

Regarding the evaluation of the cytokines, of note, Interleukin (IL)-6 was higher in patients with ICI-AIN compared with patients with ATN, but not in patients with non-ICI AIN. We did not find differences in IL-12p70.

**Figure 2:**
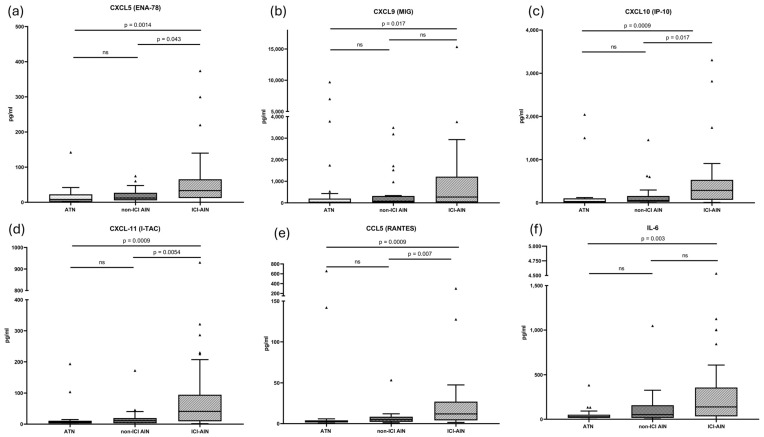
TLS and inflammation-related molecule concentrations in different study groups. *p* adjusted for multiple comparisons.

Results are summarized in Figure 2 and Table 2. Hodges–Lehmann median differences and 95% CI are shown in Supplementary Table S2.

Next, we evaluated the performance of these molecules in differentiating ICI-AIN from ATN using ROC curve analysis. Of interest, CXCL10 and CXLCL11 were the ones that showed the greatest specificity, and their PLR was over 8. Cut-off values, sensitivity, specificity, positive likelihood ratio (PLR) and area under the curve (AUC) are summarized in Table 3. ROC curves are shown in Supplementary Figure S1.

**Figure 3:**
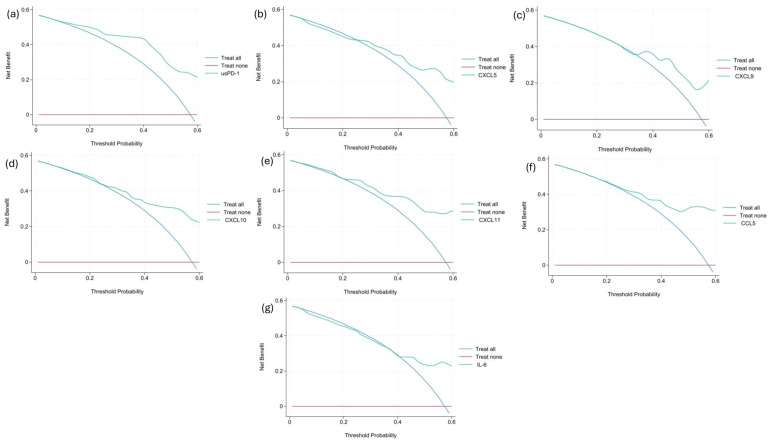
Decision curve analyses (DCAs) for (**a**) usPD-1, (**b**) CXCL5, (**c**) CXCL9, (**d**) CXCL10, (**e**) CXCL11, (**f**) CCL5 and (**g**) IL-6.

We then performed a decision curve analysis (DCA) for every statistically significant biomarker to see if there was a benefit of using these biomarkers to guide clinical decisions. For usPD-1, CXCL10, CXCL11 and CCL5, there was a net benefit of using the biomarker across the range of threshold probabilities. Graphs are shown in Figure 3.

### 2.4. Biomarker Models

We aimed to compare different models in order to determine the best at differentiating AIN from ATN. First, we performed a univariate logistic regression, summarized in Table 4. All biomarkers with significant median differences remained significant in the univariable analysis.

Then we proceeded to perform a multivariable analysis to adjust biomarkers for potential confounders (age, sex and diabetes). Hypertension was not retained in the multivariable model as it did not meet a *p* < 0.30 in the univariate analysis to maintain model parsimony. Data are summarized in Table 5.

**Figure 4:**
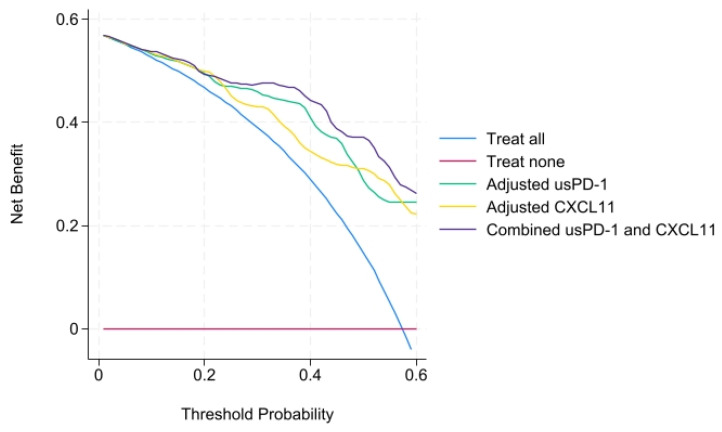
Decision curve analysis showing the net benefit of the model of usPD-1, CXCL11 and the combination of both.

All biomarkers retained statistical significance after adjustment. usPD-1 and CXCL11 were the biomarkers with higher OR, and their models were among the ones with the higher AUC. Therefore, we decided to combine both biomarkers into a single model. This model yielded the highest AUC, while also reducing the Akaike information criterion (AIC) and Bayesian information criterion (BIC) of the separate models. Furthermore, we performed a decision curve analysis for these three models (Figure 4), showing all a net benefit across the threshold.

Finally, we performed a Cox proportional hazards regression to assess the prognostic value of baseline biomarkers on survival in patients with ICI-AIN. Baseline biomarker levels, (adjusted by sex and age) were not significantly associated with the hazard of death (data summarized in Supplementary Table S3).

### 2.5. Clinical Model and Combined Model

In order to determine whether traditional biomarkers are useful by themselves or in combination with novel biomarkers, we first performed a multivariable model using the variables that met the inclusion criteria of *p* < 0.3 (creatinine, c-reactive protein and number of leukocytes in urine) and also adjusted by sex and age.

The clinical model yielded an AUC of 0.81, an AIC = 78.02 and a BIC = 92.8.

**Figure 5:**
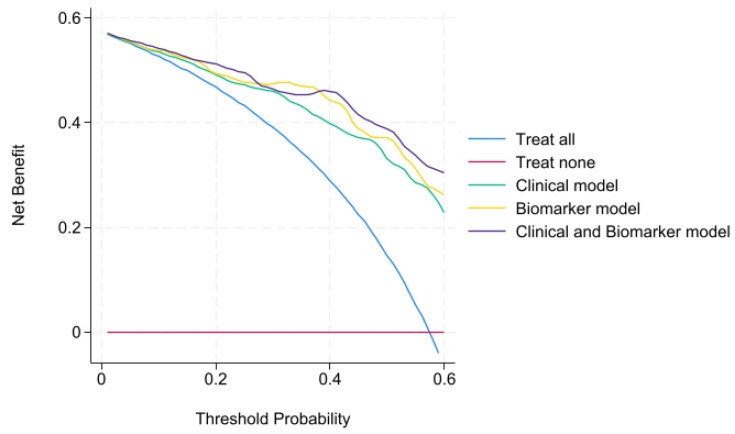
Decision curve analysis showing the performance of the clinical model, biomarker model and both compared.

Afterwards, we decided to add to the clinical model the combination of biomarkers usPD-1 and CXCL11 to see if the clinical model was improved by them. This combined model yielded the highest AUC of 0.91, while improving the AIC = 65.12 and BIC = 84.12 compared with the clinical model alone. We made the decision curve analysis to compare clinical, biomarker and combined models together, showing that all of them had a net benefit across the range of threshold possibilities (Figure 5).

### 2.6. Longitudinal Assessment of Urinary Biomarkers in ICI-AIN

Given the differences observed in the urinary levels of the studied molecules across renal diseases, we proceeded to investigate whether these molecules could serve as useful biomarkers for monitoring disease progression.

A total of 40 urine follow-up samples were available for analysis: 16 individuals had follow-up samples collected 30–90 days after diagnosis, 15 patients had samples between 91–180 days, and 9 patients had samples more than 180 days post-diagnosis (Supplementary Table S4). None of the patients had a relapse in the follow-up.

Using a linear mixed-effects model, we observed a significant decrease in urinary levels of CXCL9 (*p* = 0.036), CXCL10 (*p* = 0.036), CXCL11 (*p* = 0.018) and CCL2 (*p* = 0.036), with reductions detectable as early as 30–90 days post-diagnosis. Although all molecules tended to decrease following diagnosis and initiation of treatment, no significant changes were observed over time for PD-1, PD-L1, PD-L2, CCL3, CCL5 (*p* = 0.097) CXCL5, IL-6 or IL-12p70.

**Figure 6:**
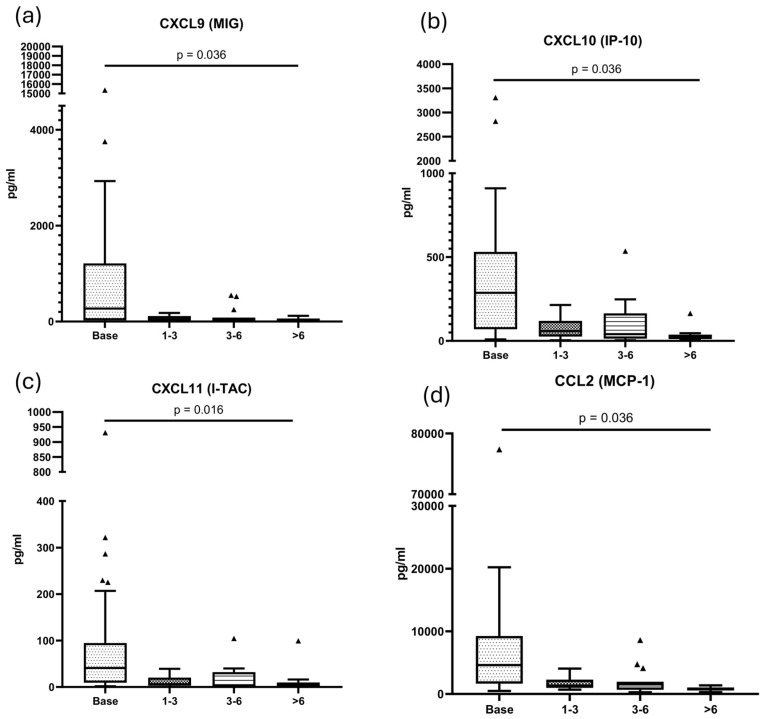
Urinary molecule concentrations during follow-up. Adjusted *p* values.

Figure 6 illustrates the evolution of the markers over time after diagnosis.

Finally, we subdivided ICI-AIN samples at diagnosis into treatment naïve (n = 25) and sampled under corticosteroid treatment (n = 10). Molecules (CXCL5, CXCL9, CXCL10, CXCL11, CCL2, CCL3, CCL5 and IL-6) were higher in the untreated group than those collected after corticosteroid treatment (multiple test adjusted *p*). This comparison is summarized in Table 6.

### 2.7. Urinary Biomarkers as Predictors of Renal Outcomes in ICI-AIN

Given the differential levels observed in ICI-AIN compared with other tubulointerstitial conditions, and the potential utility of some molecules for patient follow-up, we next sought to evaluate their value in predicting renal outcomes.

Interestingly, at the time of the diagnosis, we observed a positive correlation between serum creatinine and CCL3 (*p* = 0.008, r = 0.441) as well as CXCL9 (*p* = 0.037, r = 0.353). As expected, serum creatinine also correlated with C-reactive protein (*p* = 0.023, r = 0.436) and proteinuria (*p* = 0.036, r = 0.391).

To assess whether these molecules were associated with kidney recovery from AKI, and accounting for the severity of the initial injury, we calculated a ratio of serum creatinine at diagnosis to that at one-year follow-up. This ratio was higher in patients with elevated C-reactive protein (CRP) and leukocyturia, reflecting significant positive correlations with these parameters (CRP: r = 0.564, *p* = 0.015; leukocyturia: r = 0.464, *p* = 0.02). Regarding the novel biomarkers, the creatinine ratio also showed positive correlations with the chemokines CCL3 (r = 0.446, *p* = 0.025), CCL5 (r = 0.477, *p* = 0.016), CXCL9 (r = 0.565, *p* = 0.003), and CXCL10 (r = 0.562, *p* = 0.003).

To examine early changes in these molecules, we calculated a delta biomarker for each of them by subtracting the value at 30–90 days post-diagnosis from the value at diagnosis. For CXCL9 (r = 0.706, *p* = 0.013) and CXCL10 (r = 0.629, *p* = 0.032), the delta biomarker was positively associated with the creatinine ratio, indicating that higher early decreases in these chemokines were linked to greater changes in kidney function over the year.

## 3. Discussion

Finding non-invasive biomarkers for the diagnosis of ICI-AIN is needed for early detection and improvement of renal outcomes [24,25] as well as to avoid unnecessary toxicity associated with glucocorticoids when diagnosis may have been incorrect. This is reflected in the position statement of the American Society of Onco-Nephrology, which highlights the need to incorporate novel biomarkers into clinical decision-making, although the specific panel of biomarkers has yet to be defined [26]. In this regard, different biomarkers have been investigated in past years, such as retinol-binding protein [17], TNF-alpha [18], IL-2R [19] and others [27]. Furthermore, given that complete recovery of kidney function is not always attained [28], that the duration and dose of corticosteroid treatment are not standardized [29,30], and that re-challenge with ICI (sometimes with low-dose concomitant steroids) may lead to a new flare of ICI-AIN, the kinetics of these biomarkers can be useful for monitoring these patients.

The soluble fraction of the immune checkpoint pathway—produced by alternative splicing or direct proteolysis and detectable in serum and urine—has been of interest in the study of autoimmune diseases. Our group previously reported a significant decrease in soluble components of the immune checkpoint pathway in the urine of patients during the acute phase of ANCA-associated vasculitis [31], suggesting a potential role as a disease activity biomarker. Other authors have proposed the utility of soluble PD-1 as a marker of severity in systemic sclerosis [32], and of disease activity in systemic lupus erythematosus [33]. In cancer patients, serum soluble immune checkpoint molecule levels have been investigated as markers of disease extent and treatment response, with controversial results [34,35]. The results of the present study confirm our previous findings, showing higher usPD-1 levels in patients with ICI-AIN, with significant differences not only compared with ATN but also to non-ICI-AIN.

Chemokines (or chemotactic cytokines) are a large family of small (7–12 kDa), secreted proteins that signal through cell-surface G protein-coupled chemokine receptors. Based on the arrangement of specific cysteine residues, they are divided into four subfamilies: CC, CXC, CX3C, and XC. Their primary function is to stimulate cell migration, most notably of leukocytes [36]. They play a central role in the development and homeostasis of the immune system, as well as in immune and inflammatory responses [25,26]. CXCL9, CXCL10 and CXCL11 are mainly induced by IFN-gamma; they are produced by APCs such as macrophages, dendritic cells and stromal cells in inflammatory and tumoral environments. Elevation of chemokine levels has also been observed in the development of irAEs in patients treated with immune checkpoint blockade [28].

Specifically in renal irAEs and ICI-AIN, previous studies have reported higher levels of certain chemokines in urine—identified through proteomic analyses—as well as increased expression in renal tissue from patients with ICI-AIN [8,9,10,37]. The most consistent finding across ICI-AIN studies is the elevation of CXCL9 levels, which are significantly higher in these patients when compared with those with ATN and also with non–ICI-AIN [9,10,21]. Interestingly, the ability to distinguish ICI-AIN from non-ICI-AIN appears to be greater when measuring urinary rather than serum levels [9,10], further reinforcing the value of urinary biomarkers.

In line with the results obtained by other authors, we found higher levels of CXCL5, CXCL9, CXCL10, CXCL11, and CCL5 in ICI-AIN compared with ATN. Beyond the differences with ATN, our study also showed that urinary levels of CXCL5, CXCL10, CXCL11, and CCL5 were significantly increased in patients with ICI-AIN compared with those with AIN of other etiologies. Higher values of CXCL10 and CXCL11 suggest a more prominent activation of the interferon pathway in the context of immune checkpoint blockade. In contrast, CXCL5 is more related to the IL-1β pathway [38] and CCL5 is related to NF-κB [39] so there may be interweaving mechanisms in the pathogenesis of ICI-AIN yet to be understood.

Based on the results of this study, we propose adopting a multimarker strategy for the differential diagnosis of ICI-AIN and ATN. usPD-1 demonstrated high sensitivity, making it an excellent biomarker for ruling out false negatives and ideal for screening. In contrast, CXCL11 showed high specificity, making it valuable for confirming the diagnosis. As these molecules are related to different pathways, they could be appropriate to account for different profiles of ICI-AIN. Indeed, we have shown that a combination of both biomarkers, and after adjusting for confounders, composes a model with better performance than the models with each biomarker alone. Although the best performing model is the one that also combines traditional biomarkers, it is more mathematically complex as it involves more variables (and yields higher BIC), which could probably be addressed if the number of patients was higher.

In the prospective assessment of patients with ICI-AIN, we observed that CXCL9, CXCL10, CXCL11 and CCL2 levels decreased following treatment initiation. This decline was evident during the first follow-up period (between 30 and 90 days) and persisted in subsequent periods. Although with a small sample size, comparison of biomarkers at diagnosis showed that corticosteroid naïve patients had higher levels of biomarkers than those sampled after treatment initiation, which could mean an even faster reduction of the biomarkers once treatment is initiated. To our knowledge, the kinetics of these molecules after treatment have not been previously described, and their evaluation could be of clinical interest and serve as a reference for future studies. For example, the rapid decrease in chemokine levels may correlate with clinical observations suggesting the limited benefit of prolonged corticosteroid therapy in ICI-AIN [29].

When examining the prognostic value of the biomarkers, we found that higher urinary levels of CCL3, CCL5, CXCL9, and CXCL10 were associated with better recovery of kidney function at one-year follow-up. We hypothesize that elevated chemokine levels in urine reflect a more pronounced inflammatory milieu in the kidney, serving as a marker of patients with greater potential for functional recovery once inflammation is treated. Supporting this hypothesis, patients who exhibited the greatest reduction in CXCL9 and CXCL10 during the first follow-up period were also those who demonstrated better kidney function at one year. These biomarkers could serve as a tool to monitor local kidney inflammation and adjust immunosuppression accordingly, but more investigation is needed in this regard.

As a limitation of this study, we did not have follow-up samples for patients who, after treatment re-challenge, experienced a new episode of ICI-AIN. We speculate that these patients might exhibit a smaller decrease in biomarker concentrations or an early elevation of these molecules prior to the clinical event; further investigations are warranted. Additionally, the number of patients is relatively small, which might lead to a lack of identification of some additional differences between groups, and also to overfitting of the multivariable adjustments. Finally, we did not normalize the biomarkers to urinary creatinine, which may worsen or improve biomarker performance.

In conclusion, the evaluation of urinary soluble PD-1 (usPD-1) and chemokines can serve as useful markers for the differential diagnosis of ICI-AIN and ATN in cancer patients treated with checkpoint inhibitors, providing a non-invasive biomarker for this condition. Additionally, these molecules may be valuable for monitoring disease progression and assessing response to treatment.

## 4. Materials and Methods

### 4.1. Study Design and Population

This is a single-center, prospective observational study. We recruited all individuals diagnosed with either AIN or ATN during nephrology evaluation between 2017 and 2024 at our institution. In all cases, the diagnosis of AIN was confirmed by renal biopsy after review by a specialized pathologist. To be classified as ICI-AIN, patients were required to have received at least one dose of an ICI within the previous six months. ATN was identified either by histological confirmation (18/26, 69%) or by clinical assessment from an experienced nephrologist (8/26, 31%), the reason why each patient did not undergo a kidney biopsy is explained in Supplementary Table S5. Patients were excluded if AKI was attributed to obstructive uropathy, prerenal causes, glomerulonephritis, or unclassified etiology despite a full nephrology work-up. Further exclusions included urinary tract infection, active sepsis, patients who met clinical criteria for lupus, and positivity for anti-PR3 or MPO antibodies.

All patients included in this study provided written informed consent. This study was approved by the Bellvitge University Hospital Ethics Committee (PR143/19).

### 4.2. Biomarker Assays

Urine samples were collected at the time of the diagnosis and during routine follow-up visits in the outpatient clinics. Samples were centrifuged at 2000 rpm for 10 min and stored at −80 °C on the same day of collection.

We customized a multiplex panel (Invitrogen ProcartaPlex, Thermofisher Scientific, Waltham, MA, USA) comprising 12 molecules: sPD-1, sPD-L1, sPD-L2,CXCL5/epithelial neutrophil-activating protein 78 (ENA-78), CXCL9/monokine induced by gamma interferon (MIG), CXCL10/interferon gamma-induced protein 10 (IP-10), CXCL11/IFN-inducible T cell alpha chemoattractant (I-TAC),CCL2/monocyte chemoattractant protein-1 (MCP-1), CCL3/macrophage inflammatory protein-1 Alpha (MIP-1α), CCL5/regulated on activation, normal T-cell expressed and secreted (RANTES), IL-6 and IL-12p70. The assay was performed according to the manufacturers’ instructions with a ratio of urine of 1:2 with universal assay buffer (UAB). A Luminex MAGPIX^®^ reader (Luminex Corporation, Austin, TX, USA) was used to obtain the results. Supplementary Table S6 shows the Lower Limit of Quantification (LLOQ) and Limit of Detection (LOD) for each analyte. For values under LOD, we imputed the value as LOD/2. Measurements were performed in duplicate and the limit Coefficient of Variation (CV) for concentration was 20% in order for the assay for the sample to be considered as acceptable (otherwise it would be repeated for that sample), with a mean intra-assay CV of 4.41% and inter-assay CV of 16.9%. The maximum number of freeze–thaw cycles was two.

### 4.3. Clinical Data Collection

Samples and data from patients included in this study were provided by the Biobank HUB-ICO-IDIBELL (PT20/00171), integrated in the Spanish National Biobanks Network and they were processed following standard operating procedures with the appropriate approval of the ethics and scientific committees.

The main demographic variables of interest (age, sex), and prior medical conditions (hypertension, diabetes, chronic kidney disease) were recorded from medical records. Baseline central laboratory data were obtained, including complete blood count, serum creatinine, CRP, proteinuria, leukocyturia, and hematuria. Furthermore, creatinine at one-year follow-up was also recorded. For patients with AIN, the timing of corticosteroid initiation relative to the sample at diagnosis was recorded.

For ICI-AIN patients, we also recorded the type of neoplasm, the specific ICI and the presence of extrarenal irAEs (rash, fever, arthralgias or other irAEs). For patients with non-ICI AIN, data regarding the etiology of the AIN were recorded.

Biopsy specimens were stained with hematoxylin and eosin (H&E), periodic acid-Schiff (PAS), Jones methenamine silver, and Masson’s trichrome, and were evaluated by an experienced nephropathologist.

### 4.4. Statistical Methods

The statistical analysis was performed with GraphPad Prism 8.0 (GraphPad Software, La Jolla, CA, USA), IBM SPSS Statistics 26.0 (IBM Corp., Armonk, NY, USA) and STATA 19.0 BE (StataCorp, College Station, TX, USA). Categorical data are presented as number (n) and proportions (%). Continuous variables are shown as mean ± standard deviation. Comparisons between two groups were performed using Student’s *t* test for normally distributed quantitative data, while the Mann–Whitney U test was applied when normality was not met. For qualitative variables, either the chi-square test or Fisher’s exact test was employed. We reported effect sizes using the Hodges–Lehmann estimator with its corresponding 95% confidence interval.

ROC curves were plotted to assess the accuracy of particular biomarkers. The optimal cutoff—defined as the point with the highest combined sensitivity and specificity—was determined using the Youden index.

To evaluate the clinical utility of the biomarkers and predictive models we performed DCAs. We assessed the net benefit across a range of threshold probabilities from 5% to 60%.

We performed univariable logistic regressions of the variables and biomarkers to determine their OR of having ICI-AIN compared with ATN; as well as multivariate analysis adjusting for possible confounders (age, sex, and clinical variables with *p* < 0.3 in the univariable analysis). For comparison of models, AUC, AIC and BIC were calculated.

For survival analysis, we performed a Cox proportional hazards regression model.

Due to the skewed distribution of biomarkers and analytic values (creatinine, c-reactive protein, urinary leukocytes and blood eosinophils), in order to comply with the linearity assumption, values were log-transformed (base 10) for logistic regressions, Cox regressions and DCAs. To account for zero values, a log(x + 1) transformation was applied. Missing values in CRP were handled using multiple imputation by chained equations (MICE) to minimize selection bias and preserve statistical power; 20 imputed datasets were generated.

*p* values for comparison of medians, ROC curves, odds ratios and hazard ratios where all biomarkers were present were adjusted using the Benjamini and Hochberg (BH) method to control false discovery rate (FDR).

Spearman’s correlation was applied to analyze non-normally distributed variables. For analysis of follow-up samples, a linear mixed-effects model was used.

## Figures and Tables

**Table 1:** Main characteristics at diagnosis of the cohort. ATN, acute tubular necrosis. AIN, Acute interstitial nephritis, IQR: Interquartile range.

	ATN n = 26	AIN n = 64	*p* Value
Age	66.06 ± 11.09	67.03 ± 11.06	*p* = 0.71
Sex (male)	20/26 (76.92%)	37/64 (57.81%)	*p* = 0.089
Creatinine (umol/L, median [IQR])	239.5 [165.8–435.3]	220.5 [220.5–364.8]	*p* = 0.74
Hypertension (% patients)	16/26 (61.54%)	38/64 (59.38%)	*p* = 0.85
Diabetes (% patients)	9/17 (34.62%)	14/64 (21.88%)	*p* = 0.21
Pre-existing CKD (% patients)	8/26 (30.77%)	15/64 (23.44%)	*p* = 0.47
C-reactive protein (median [IQR])	7.7 [5.2–17.9]	32.2 [7–77.2]	*p* = 0.015
Leukocyturia (% patients)	16/26 (61.54%)	47/64 (73.44%)	*p* = 0.26
Leukocyturia (Leukocytes/μL, median [IQR])	13 [4–24]	17 [9–43]	*p* = 0.02
Microhaematuria (% patients)	8/26 (30.77%)	11/64 (17.19%)	*p* = 0.15
Eosinophilia (% patients)	0/26 (0%)	15/64 (23.44%)	*p* = 0.0045

**Table 2:** This table summarizes the urinary levels of the evaluated molecules and the differences among groups. Data are shown as median [IQR]. *p* adjusted for multiple comparisons.

Molecule	ICI-AIN (pg/mL)	ATN (pg/mL)	Non-ICI AIN (pg/mL)	*p*-Value ICI-AIN vs. ATN	*p*-Value ICI-AIN vs. Non-ICI AIN	*p*-Value ATN vs. Non-ICI AIN
CXCL5	33.3 [12.17–65.45]	8.09 [1.9–22.56]	12.23 [5.58–26.99]	0.001	0.043	0.2
CXCL9	270.2 [25.98–1213]	16.4 [6.04–198.8]	76.45 [11.47–320.6]	0.017	0.22	0.47
CXCL10	287.1 [69.28–531]	30.2 [12.52–103.9]	54.59 [22.51–158.3]	0.0009	0.017	0.32
CXCL11	41.08 [9.25–94.78]	6.19 [2.38–10.77]	11.18 [2.84–19.65]	0.0009	0.005	0.2
CCL5	11.94 [4.05–26.99]	2.26 [1.43–10.45]	4.95 [2.21–8.51]	0.0009	0.007	0.11
IL-6	138.8 [32.85–356.4]	29.12 [14.91–51.03]	51.89 [14.75–157.2]	0.0025	0.19	0.21

**Table 4:** Univariable analysis. *p* adjusted for multiple comparisons (*: *p* < 0.05). Continuous variables log-transformed.

Variables	*p*-Value	OR (95% CI)
Age	0.94	1.002 (0.95–1.06)
Sex (Ref female)	0.67	0.63 (0.23–2.41)
Hypertension	0.37	0.32 (0.21–1.66)
Diabetes	0.072	0.24 (0.065–0.91)
Creatinine (log)	0.105	0.09 (0.008–1.08)
C-reactive protein (log)	0.04 *	3.37 (1.24–9.16)
Eosinophils (log)	0.67	1.13 (0.63–2.05)
Leukocytes (log)	0.026 *	
usPD-1 (log)	0.001 *	8.59 (2.63–27.97)
usPD-L1 (log)	0.35	2.1 (0.54–8.17)
usPD-L2 (log)	0.23	1.67 (0.84–3.30)
CXCL5 (log)	0.016 *	4.11 (1.58–10.68)
CXCL9 (log)	0.033 *	2.01 (1.15–3.5)
CXCL10 (log)	0.007 *	4.95 (1.95–12.56)
CXCL11 (log)	0.001 *	7.59 (2.42–23.71)
CCL2 (log)	0.26	2.05 (0.71–5.91)
CCL3 (log)	0.57	1.32 (0.6–2.91)
CCL5 (log)	0.015 *	5.85 (1.79–19.16)
IL-6 (log)	0.026 *	3.11 (1.33–7.28)
IL-12p70 (log)	0.26	4.73 (0.51–43.98)

**Table 6:** Differences between diagnosis samples according to corticosteroid initiation before treatment (adjusted *p* values for multiple comparisons).

Molecule	Prior to Corticosteroids (pg/mL)	After Corticosteroids (pg/mL)	Adjusted *p*
CXCL5	38.5 [19.1–78.96]	12.62 [4.75–30.82]	0.039
CXCL9	557.3 [62.22–1569]	23.81 [10.10–477.9]	0.039
CXCL10	361.8 [113.1–629.5]	82.48 [15.16–309]	0.03
CXCL11	51.86 [11.7–158.1]	9.87 [6.61–43.73]	0.032
CCL2	5715 [2771–13311]	1874 [1126–4621]	0.039
CCL3	7.64 [3.54–28.98]	1.13 [1.13–3.64]	0.01
CCL5	22.59 [7.25–28.43]	3.86 [1.76–9.61]	0.01
IL-6	172.1 [65.97–581]	29.6 [18.51–111.4]	0.032

**Table 3:** Statistics and optimum cutoff values of biomarkers. PLR, positive likelihood ratio. AUC, area under the curve. *p* adjusted for multiple comparisons.

	Cut-Off Value	Sensitivity (%)	Specificity (%)	PLR	AUC	95% CI	*p*-Value
CXCL5	>11.91	80	65.38	2.31	0.76	0.6503–0.8892	0.001
CXCL9	>19.59	88.57	51.54	2.3	0.71	0.5705–0.8537	0.013
CXCL10	>129.2	62.86	92.31	8.17	0.78	0.6631–0.9050	0.0009
CXCL11	>15.77	62.86	92.31	8.17	0.81	0.6935–0.9175	0.0009
CCL5	>5.04	74.29	84.62	4.83	0.8	0.6822–0.9233	0.0009
IL-6	>48.56	71.43	76.92	3.095	0.76	0.6422–0.8831	0.0015

**Table 5:** Logistic regression of each biomarker; adjusted by age, sex and diabetes. AIC, Akaike information criterion. BIC, Bayesian information criterion.

Variables	*p*-Value	OR (95% CI)	AUC	AIC/BIC
usPD-1 (log)	0.001	9.54 (2.53–36.01)	0.81	72.24/82.8
CXCL5 (log)	0.011	3.82 (1.35–10.81)	0.75	80.34/90.9
CXCL9 (log)	0.015	2.01 (1.15–3.53)	0.75	81.79/92.35
CXCL10 (log)	0.001	4.91 (1.86–12.97)	0.81	74.42/84.97
CXCL11 (log)	0.001	7.02 (2.16–22.76)	0.82	71.44/82
CCL5 (log)	0.007	6.57 (1.68–25.77)	0.81	78.69/89.24
IL-6 (log)	0.013	3.01 (1.26–7.18)	0.77	80.52/91.08
usPD-1 (log) + CXCL11 (log)	-	-	0.86	67.34/80.02

## Data Availability

Data are publicly available under CC BY 4.0 at https://doi.org/10.6084/m9.figshare.31082386.v1.

## Supplementary Materials

The supporting information can be downloaded at https://www.mdpi.com/article/10.3390/ijms27031240/s1.

## Abbreviations

The following abbreviations are used in this manuscript:

ICIs	Immune Checkpoint Inhibitors
TCR	T Cell Receptor
MHC	Major Histocompatibility Complex
APCs	Antigen-Presenting Cells
PD-L1	Programmed Cell Death Ligand 1
PD-L2	Programmed Cell Death Ligand 2
PD-1	Programmed Death 1
NK	Natural Killer (cells)
irAEs	Immune-Related Adverse Events
ICI-AKI	ICI-associated Acute Kidney Injury
ICI-AIN	ICI-associated Acute Interstitial Nephritis
AIN	Acute Interstitial Nephritis
C-X-C/C-C	Chemokine subfamilies (based on cysteine residues)
TLS	Tertiary Lymphoid Structures
AKI	Acute Kidney Injury
ATN	Acute Tubular Necrosis
eGFR	Estimated Glomerular Filtration Rates
usPD-1	Urinary Soluble Programmed Death 1
usPD-L1	Urinary Soluble Programmed Cell Death Ligand 1
usPD-L2	Urinary Soluble Programmed Cell Death Ligand 2
ROC	Receiver Operating Characteristic
AUC	Area Under the Curve
CI	Confidence Interval
PLR	Positive Likelihood Ratio
CXCL	C-X-C Motif Chemokine Ligand
CCL	C-C Motif Chemokine Ligand
IL-6	Interleukin-6
IL-12p70	Interleukin 12p70
DCA	Decision Curve Analysis
OR	Odds Ratio
AIC	Akaike Information Criterion
BIC	Bayesian Information Criterion
IQR	Interquartile Range
CRP	C-Reactive Protein
IFN-gamma	Interferon-gamma
NF-κB	Nuclear Factor kappa-light-chain-enhancer of activated B cells
ANCA	Anti-Neutrophil Cytoplasmic Antibody
TNF-alpha	Tumor Necrosis Factor-alpha
IL-2R	Interleukin-2 Receptor
MICE	Multiple Imputation by Chained Equations
BH	Benjamini and Hochberg (method)
FDR	False Discovery Rate
PR3	Proteinase 3
MPO	Myeloperoxidase
ENA-78	Epithelial Neutrophil-Activating Protein 78
MIG	Monokine Induced by Gamma interferon
IP-10	Interferon gamma-induced Protein 10
I-TAC	IFN-inducible T cell Alpha Chemoattractant
MCP-1	Monocyte Chemoattractant Protein-1
MIP-1α	Macrophage Inflammatory Protein-1 Alpha
RANTES	Regulated on Activation, Normal T-cell Expressed and Secreted
UAB	Universal Assay Buffer
LLOQ	Lower Limit of Quantification
LOD	Limit of Detection
CV	Coefficient of Variation
H&E	Hematoxylin and Eosin
PAS	Periodic Acid-Schiff
