# Supplementary material for: Urinary Chemokines in the Diagnosis and Monitoring of Immune Checkpoint Inhibitor-Associated Nephritis
Source: Int J Mol Sci. 2026 Jan 26;27(3):1240. doi: 10.3390/ijms27031240 (PMC12898666; doi:10.3390/ijms27031240)
Supplement: Supplementary file 1 [file ijms-27-01240-s001.zip › Supplementary Table S6.pdf]

	LLOQ	LOD
**PD-1**	7.69	7.69
**PD-L1**	3.88	2.8
**PD-L2**	62.65	17.19
**CXCL5**	4.64	0.2
**CXCL9**	0.76	0.76
**CXCL10**	1.57	1.33
**CXCL11**	1.44	0.15
**CCL2**	4.71	4.71
**CCL3**	1.27	0.28
**CCL5**	0.84	0.53
**IL-6**	7.93	0.2
**IL12p70**	7.81	4.11

Supplementary Table S6. Lower limit of quantification (LLOQ) and Limit of Detection (LOD) for each analyte.
