# Supplementary material for: Urinary Chemokines in the Diagnosis and Monitoring of Immune Checkpoint Inhibitor-Associated Nephritis
Source: Int J Mol Sci. 2026 Jan 26;27(3):1240. doi: 10.3390/ijms27031240 (PMC12898666; doi:10.3390/ijms27031240)
Supplement: Supplementary file 1 [file ijms-27-01240-s001.zip › Supplementary Table S1.pdf]

	ICI-AIN<br>N = 35	non-ICI AIN<br>N = 29	p-value
**Age**	66.28 ± 9.006	68.01 ± 13.24	p = 0.54
**Sex (Male)**	25/35 (71.43%)	12/29 (41.38%)	p = 0.022
**Creatinine (µmol/L, median [IQR])**	197 [151-265]	290 [192-454]	p = 0.003
**Hypertension (% patients)**	17/35 (48.57%)	21/29 (72.41%)	p = 0.022
**Diabetes (%patients)**	4/35 (11.43%)	10/29 (28.57%)	p = 0.13
**Pre-existing CKD (%patients)**	7/35 (20%)	8/29 (27.59%)	p = 0.56
**Cancer type**	Lung 19/35 (54.29%)<br>Kidney 5/35 (14.29%)<br>ENT 2/35 (5.71%)<br>Breast 2/35 (5.71%)<br>Mesothelioma 2/35 (5.71%)<br>Endometrium 1/35 (2.86%)<br>Digestive 1/35 (2.86%)<br>Thyroid 1/35 (2.86%)<br>Hematological 1/35 (2.86%)<br>Unknown 1/35 (2.86%)	-	-
**C- Reactive protein (mg/L, median [IQR]))**	37 [12-91]	24 [3.8-52]	p = 0.23
**Leukocyturia (%patients)**	28/35 (80%)	19/29 (65.52%)	p = 0.26

**Microhaematuria (%patients)**	6/35 (17.14%)	5/29 (17.24%)	p = 0.99
**Eosinophilia (%patients)**	10/35 (28.57%)	5/29 (17.24%)	p = 0.38
**Etiology**	Anti PD-1 22/35 (62.86%)<br>Anti PD-L1 8/35 (22.86%)<br>Anti PD-1 + Anti CTLA4 2/35 (5.71%)<br>Anti PD-L1 + Anti CTLA4 1/35 (2.86%)<br>Anti PD-1 + Clinical Trial 2/35 (5.71%) (vibostolimab, MK-4830)	**Drug-induced** 16/29 (55.17%)<br>**Unknown** 7/29 (24.14%)<br>**Immunologic** 4/29 (13.79%)<br>**Sarcoidosis** 2/29 (6.9%)	-
**Extrarenal irAEs**	Overall 15/35 (42.86%)<br>Fever 6/35 (17.14%)<br>Dermatologic 5/35 (14.29%)<br>Endocrine 4/35 (11.42%)<br>Gastrointestinal 3/35 (8.57%)<br>Musculoskeletal 3/35 (8.57%)	-	-

Supplementary Table S1. Baseline characteristics of AIN subgroups
