# Supplementary material for: Urinary Chemokines in the Diagnosis and Monitoring of Immune Checkpoint Inhibitor-Associated Nephritis
Source: Int J Mol Sci. 2026 Jan 26;27(3):1240. doi: 10.3390/ijms27031240 (PMC12898666; doi:10.3390/ijms27031240)
Supplement: Supplementary file 1 [file ijms-27-01240-s001.zip › Supplementary Table S2.pdf]

| Molecule | ICI-AIN vs ATN | p-value ICI-AIN vs non-ICI AIN | p-value ATN vs non-ICI AIN |
|---|---|---|---|
| **CXCL5** | 19,67 (7.57 to 34.55) | 13,02 (1.930 to 29.50) | 5,165 (-0.6400 to 12,27) |
| **CXCL9** | 164,4 (11,36 to 538,7) | 68,26 (5.900 to 480.9) | 16,30 (-6,600 to 115,5) |
| **CXCL10** | 172,5 (54,28 to 348,3) | 121,5 (27.54 to 313.3) | 17,42 (-4,850 to 51,74) |
| **CXCL11** | 34,27 (6,820 to 49,06) | 27,97 (5,520 to 46,01) | 3,020 (0,2100 to 8,510) |
| **CCL5** | 7,845 (2,760 to 17,40) | 6,810 (1,680 to 14,58) | 1,315 (0,06000 to 3,780) |
| **IL-6** | 81,74 (24,16 to 156,4) | -43,98 (-132,1 to 2,580) | 21,47 (-2,730 to 62,56) |

**Supplementary Table S2.** *Hodges-Lehmann estimator of the median differences (95% CI)*
