# Supplementary material for: Urinary Chemokines in the Diagnosis and Monitoring of Immune Checkpoint Inhibitor-Associated Nephritis
Source: Int J Mol Sci. 2026 Jan 26;27(3):1240. doi: 10.3390/ijms27031240 (PMC12898666; doi:10.3390/ijms27031240)
Supplement: Supplementary file 1 [file ijms-27-01240-s001.zip › Supplementary Table S3.pdf]

Biomarker	Hazard Ratio (HR)	p- value	95% CI
**PD1**	.3980712	0.092	.1361583 1.163798
**PDL1**	0.497	0.196	.1723619 1.432966
**PDL2**	1.401989	0.324	.715892 2.745626
**CXCL5**	.8592117	0.570	.5093264 1.449453
**CXCL11**	.8559042	0.648	.4385429 1.670468
**IL6**	.9435135	0.784	.6230419 1.428825
**IL12p**	.2670898	0.111	.0526545 1.354811
**CXCL10**	.8011082	0.533	.3989555 1.608637
**CCL2**	.8994048	0.828	.3457289 2.339778
**CXCL9**	1.024818	0.921	.6309545 1.664546
**CCL3**	.7586591	0.445	.3731692 1.542366
**CCL5**	.8586376	0.713	.380729 1.936439

Supplementary Table S3. Hazard ratios for death according to the biomarkers, adjusted by sex and age. Log-transformed variables. P-values corrected for multiple comparisons.
