# Supplementary material for: Urinary Chemokines in the Diagnosis and Monitoring of Immune Checkpoint Inhibitor-Associated Nephritis
Source: Int J Mol Sci. 2026 Jan 26;27(3):1240. doi: 10.3390/ijms27031240 (PMC12898666; doi:10.3390/ijms27031240)
Supplement: Supplementary file 1 [file ijms-27-01240-s001.zip › Supplementary Table S4.pdf]

	30-90 days	91-180 days	More than 180 days
**ICI-AIN = 40**	16	15	9

Supplementary Table S4. Follow up samples from patients with ICI-AIN and the distribution of time period obtained.
