# Supplementary material for: Urinary Chemokines in the Diagnosis and Monitoring of Immune Checkpoint Inhibitor-Associated Nephritis
Source: Int J Mol Sci. 2026 Jan 26;27(3):1240. doi: 10.3390/ijms27031240 (PMC12898666; doi:10.3390/ijms27031240)
Supplement: Supplementary file 1 [file ijms-27-01240-s001.zip › Supplementary Table S5.pdf]

Patient	Baseline characteristics	Etiology of ATN	Reason for not performing biopsy
1	Hypertension, Diabetes	AKIN 2 after Cisplatin initiation.	Spontaneous kidney function improvement
2	CKD, hypertension	AKIN 2 during bacteremia	Spontaneous kidney function improvement
3	CKD, hypertension	AKIN after Cisplatin initiation	Spontaneous kidney function improvement
4	Hypertension, Diabetes	AKIN 3 after maintained dehydration due to diarrhea	Spontaneous kidney function improvement
5	Diabetes	AKIN 3 after septic shock and need of ICU	Spontaneous kidney function improvement
6	CKD	AKIN 3 after Gentamicin treatment	Spontaneous kidney function improvement
7	Hypertension	AKIN 2 after carboplatin initiation	Spontaneous kidney function improvement
8	Hypertension	AKIN 3 due to maintained hypotension after suspension of hormonal treatment in patient with panhypopituitarism and diarrhea	Spontaneous kidney function improvement

Supplementary Table S5. Patients with clinically diagnosed ATN and the reason for not performing kidney biopsy.
