# Supplementary figures and images for: Urinary Chemokines in the Diagnosis and Monitoring of Immune Checkpoint Inhibitor-Associated Nephritis
Source: Int J Mol Sci. 2026 Jan 26;27(3):1240. doi: 10.3390/ijms27031240 (PMC12898666; doi:10.3390/ijms27031240)

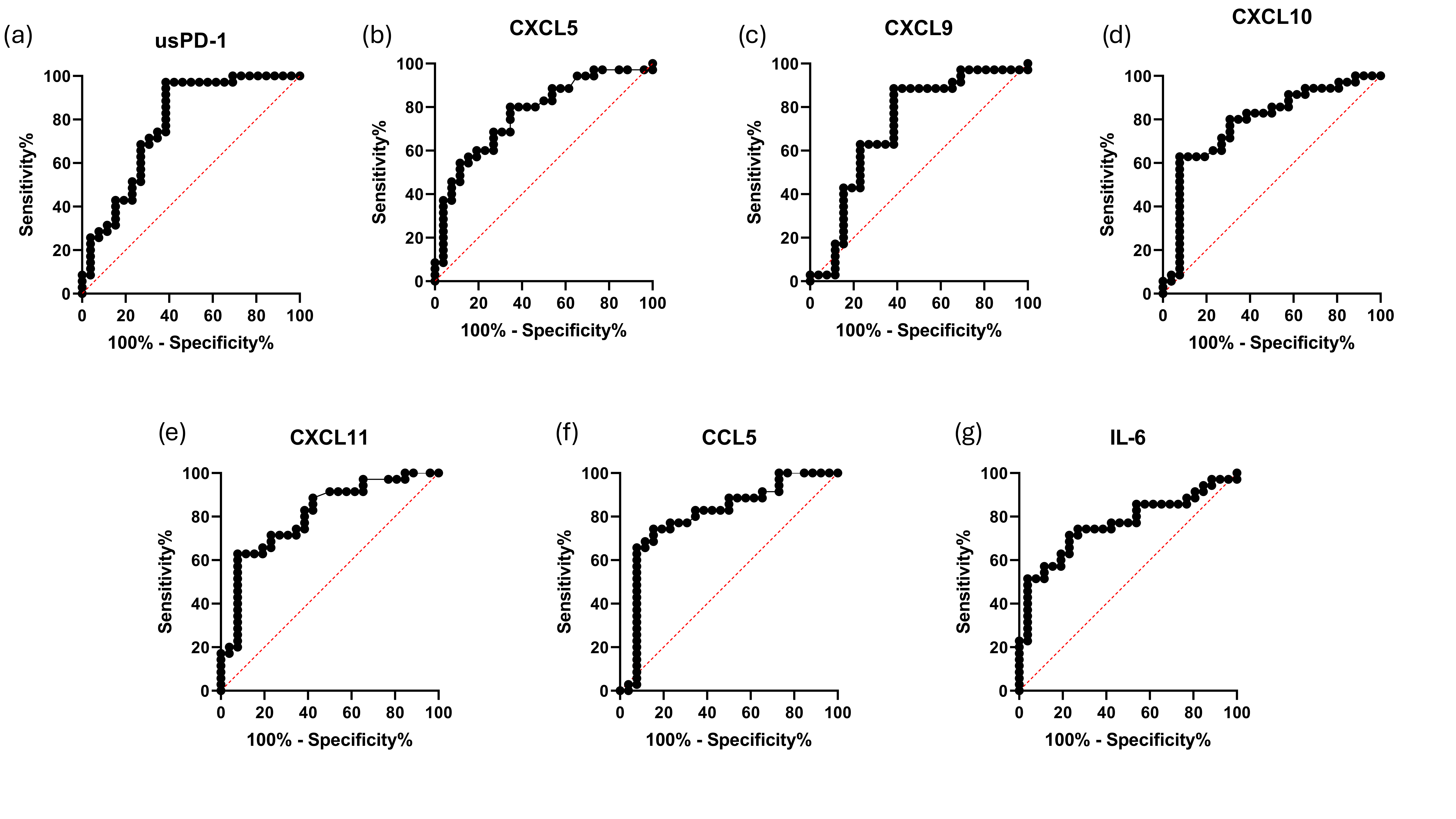

Supplement: Supplementary file 1 [file ijms-27-01240-s001.zip › Supplementary Figure S1. ROC curves for differentiating ICI-AIN from ATN.png]
